# Rocovery from a Scalp Wound

**Published:** 1871-03

**Authors:** 


### Abridgments from our Exchanges.

Recovery from a Scalp Wound.—Dr. R. C. Moore reports a case of a man who lost, by the scalping knife, all the tissues above the pericranium, and a part of this membrane, over a space of 9 by 7 inches. When presented for treatment, the remaining pericranium was dried to the bone; this was two days after the scalping. The only dressing applied was pure olive oil, on surgeon's lint. In about three weeks the external table of the skull began to esfoliate. As this process progressed, granulation sprung up from the diplœ, and in there the entire surface presented the appearance of a healthy wound. The suppuration was not profuse, but the patient being naturally strong, bore it well, and in about three months from the time of injury, nearly the whole surface was covered with a cicatrix. *Medical and Surgical Reporter.*

